# Effect of *Nosema ceranae* infection and season on the gut bacteriome composition of the European honeybee (*Apis mellifera*)

**DOI:** 10.1038/s41598-022-13337-4

**Published:** 2022-06-04

**Authors:** Clara Jabal-Uriel, Claudio Alba, Mariano Higes, Juan Miguel Rodríguez, Raquel Martín-Hernández

**Affiliations:** 1 Laboratorio de Patología Apícola, Centro de Investigación Apícola y Agroambiental (CIAPA), IRIAF – Instituto Regional de Investigación y Desarrollo Agroalimentario y Forestal, Consejería de Agricultura de la Junta de Comunidades de Castilla-La Mancha, 19180 Marchamalo, Spain; 2 Department of Nutrition and Food Science, Complutense University of Madrid, 28040 Madrid, Spain; 3 Instituto de Recursos Humanos para la Ciencia y la Tecnología (INCRECYT – ESF/EC-FSE), Fundación Parque Científico y Tecnológico de Castilla – La Mancha, 02006 Albacete, Spain

**Keywords:** Microbial communities, Parasitology, Microbiology, Fungi, Fungal host response

## Abstract

*Nosema ceranae* is an intracellular parasite that infects honeybees’ gut altering the digestive functions; therefore, it has the potential of affecting the composition of the gut microbiome. In this work, individual bees of known age were sampled both in spring and autumn, and their digestive tracts were assessed for *N. ceranae* infection. Intestinal microbiome was assessed by sequencing the bacterial 16S rRNA gene in two different gut sections, the anterior section (AS; midgut and a half of ileum) and the posterior section (PS; second half of ileum and rectum). A preliminary analysis with a first batch of samples (n = 42) showed that AS samples had a higher potential to discriminate between infected and non-infected bees than PS samples. As a consequence, AS samples were selected for subsequent analyses. When analyzing the whole set of AS samples (n = 158) no changes in α- or β-diversity were observed between infected and non-infected bees. However, significant changes in the relative abundance of Proteobacteria and Firmicutes appeared when a subgroup of highly infected bees was compared to the group of non-infected bees. Seasonality and bees’ age had a significant impact in shaping the bacteriome structure and composition of the bees’ gut. Further research is needed to elucidate possible associations between the microbiome and *N. ceranae* infection in order to find efficient strategies for prevention of infections through modulation of bees’ microbiome.

## Introduction

As a result of their interactions within the colony, eusocial insects have developed specialized and characteristic microbiomes that play an important role in shaping their ecology and evolution^1,2^. The European honeybee (*Apis mellifera*) has been studied as a model for gut microbiota research^3,4^ because of their relatively simple and ubiquitous microbiota^1,2^. Their gut bacteriome seems to be dominated by a few genera belonging to the phyla Proteobacteria, Firmicutes and Actinobacteriota, which account for more than 95% of the gut bacteriome in worker bees^5,6^.

In adult bees, this core bacteriome is acquired through horizontal transmission after adult honeybees emerge from their cells and by contacting with older bees and other elements of the hive, such as bee bread and the combs^7^, and it is well established around day 4 post emergence^3,8^. The digestive system of bees is formed by well differenced anatomical parts. The crop is the portion with fewer bacteria (< 1%), followed by the ventriculus (1–4%), the ileum (4–10%) and, finally, the rectum, that harbours up to 90% of the total gut bacteria^8^. The bacteriome of the digestive tract seems to be very stable in healthy bees although its composition is usually characterized by a certain degree of inter-individual variability, even among honeybees with the same age and belonging to the same colony^5,7^. In fact, some studies have found that there are differences in the microbial profile of honeybees depending on their developmental stage, their age and/or in-hive tasks^9–11^. Season is another factor that influences the composition of their microbiota, probably because of the season-associated meteorological conditions and dietary changes^12–15^.

The gut microbiota is involved in growth and development of the honeybees and it contributes to host health by participating in food digestion, modulation of the immune system and defence against pathogens^16,17^. If it is disrupted, it may have consequences to the ability of bees to cope with environmental stressors, such as parasites^17,18^. Pathogenic organisms, including trypanosomatids, viruses and fungi, may share the digestive niche with the gut bacteriome^19^. Among fungi, the microsporidia *Nosema ceranae* is an obligate intracellular parasite^20^ with a high prevalence on honeybee colonies worldwide. This parasite infects the epithelial cells of the ventriculus (or midgut), causing extensive destruction of the tissue and damaging the peritrophic membranes^21,22^ and exerting deleterious immunomodulatory roles^23,24^, leading to a shortening of the bee lifespan^20,25,26^. The lesions on the ventriculus have been reported to reflect into changes of the metabolism of carbohydrates^27–30^. Therefore, the infection by *N. ceranae* could modify the state in which food reaches the posterior parts of the digestive tract of the bees, a fact that may contribute to the modifications of the intestinal microbiota increasing or decreasing some core bacteria that have been reported previously^31–35^. Consequently, the objective of this study was to determine if the infection by *N. ceranae* modulates the gut bacteriome composition by analysing naturally infected or non-infected adult honeybees in two different seasons (spring and autumn).

## Material and methods

### Experimental design and sample selection

**Figure 1:**
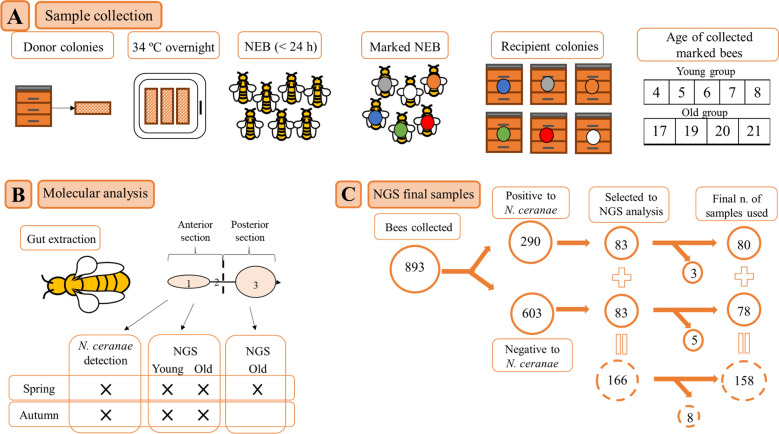
Experimental design. (**A**) Sample collection. In both seasons, capped brood frames were taken from donor colonies and put in an incubator overnight to obtain newly emerged bees (NEB). The bees were marked and distributed in groups in recipient colonies. The collected bees were grouped in young and adult bees and analyzed in the laboratory. (**B**) Molecular analysis. The gut (from midgut to rectum) was extracted and cut in two: anterior section (ventriculum (1) and half of the ileum (2)) and posterior section (half of the ileum (2) and rectum (3)), indicated by the dashed line. Samples were assessed for *N. ceranae* and metataxonomic analyses as indicated. (**C**) Next generation sequencing (NGS) final samples. Selection of samples from the total number of bees collected towards the final samples used for the metataxonomic analyses. In the process of DNA extraction and sequencing, 8 samples were discarded (3 positive and 5 negative to infection).

The experimental design is shown in Fig. 1. Honeybees were collected in spring (June 2019) and autumn (October 2019) from experimental colonies of an apiary located 16 km away from the Centro de Investigación Apícola y Agroambiental (CIAPA, Marchamalo, Spain, 40° 40′ 55,77″ N; 3° 12′ 32,72″ W). In order to avoid genetic homogeneity, capped brood combs from 8 and 6 donor colonies of *A. mellifera* subsp. *iberiensis* were collected in spring and autumn, respectively. Combs were brushed to remove the remaining adult bees and transported to the CIAPA laboratory. They were placed overnight in an incubator (Memmert ® IPP 500) at 34 ± 1 °C in order to have newly emerged adult bees of less than 24 h the following day.

The next day (day 0), the newly emerged bees were allotted in 6 groups of approximately 300 bees each (1800 bees in total), one for each recipient colony, marked in the thorax with enamel paint (Posca PC-5M, Mitsubishi Pencil Co), and afterwards introduced in 6 recipient colonies, previously established, known to be infected by *N. ceranae* (PCR tested^36^).

All colonies in this study were located in Langstroth hives and were treated against *Varroa destructor* according to the current Spanish legislation (i. e. 2 strips of Amitraz per colony applied during 6 weeks in October 2018 and 2019 after the experiment was completed).

From each colony, 15 young (4 to 8 days post emergence [p. e.]) and 15 old bees (17 to 21 days p. e) were collected daily (Table 1) and immediately carried to the laboratory for analysis. Once there, the bees were anesthetized in cold and processed individually in a laminar flow cabinet (Telstar AV–30/70) where the guts (from midgut to rectum) were carefully removed from each sample by pulling the last segment of the abdomen with sterile tweezers. Every gut was cut, separating the ventriculum from the rectum by an incision in the middle of the ileum (Fig. 1) which allowed us to obtain the two sections by cutting without using a stereo microscope. In this way, the resulting sections were: (i) anterior section (AS), containing the ventriculum and the first half of the ileum, and (ii) posterior section (PS), containing the second half of the ileum and the rectum. Each resulting section was placed separately in 96-well plates (Qiagen®) containing 250 µL of sterile PBS buffer and 4 glass beads (2 mm diameter, Sigma ®). The tissues were homogenized for 2 min at 30 Hz (TyssueLyser II, QIAgen®). Controls containing only PBS buffer and the homogenizing reagents but devoid of biological samples were also included in the plates to be used as quality controls during DNA extraction and metataxonomic analysis.

**Table 1:** Honeybees analyzed in this study.

	Young					Old				Total
	4	5	6	7	8	17	19	20	21	
**Spring**										
Total n. of bees	–	–	90	45	45	70	40	35	30	335
PCR analysis (P–N)			6–84	8–37	4–41	46–24	34–6	27–8	25–5	150–205
Selected for NGS analysis (P–N)			5–5	7–7	3–3	3^†^–7^†^	3^†^–3^†^	10^†^–8^†^	7^†^–5^†^	38–38
**Autumn**										
Total n. of bees	45	90	90	90	–	–	90	90	43	538
PCR analysis (P–N)	1–44	4–86	8–82	5–85			44–46	49–41	30–13	140–398
Selected for NGS analysis (P–N)	1–1	4–4	8–8	2–2			10–10	10–10	10–10	45–45

Includes the total number of samples collected and analyzed by PCR to detect *N. ceranae* each day and the results (P—positive and N—negative). It also includes the number of samples selected for the next generation sequencing (NGS) analysis in each sample set, which are infected (P) and non-infected (N) bees in the group of young and old bees (with the age expressed in days) from both seasons, spring and autumn. The numbers correspond to anterior section samples analyzed.

^†^In old adult bees from spring, the posterior section from the samples was analyzed as well.

### Detection and quantification of *Nosema* spp

Detection of *N. ceranae* was performed using the AS since ventriculum is the target for infection. For this purpose, 50 µL of homogenized AS samples were transferred to a 96-well plate (Qiagen®) and 50 µL of Tris–HCl lysis solution were added and incubated at 95 °C for 15 min. A triplex conventional PCR using gelified plates (BioTools®) in a Mastercycler® ep gradient S (Eppendorf) was performed to assess *Nosema* spp. following the protocol described in^36,37^. The resulting amplicons were analyzed in a QIAxcel Advanced System (QIAgen®). Non-template controls (NTC) and a positive control of *N. ceranae* and *N. apis* were also included in the reaction plates.

Subsequently, samples from those bees that were positive to *N. ceranae* (by triplex conventional PCR) were analyzed by real time quantitative PCR (RT-qPCR) in order to quantify the copy load of the *polar tubule protein-3* (PTP-3) gene of *N. ceranae*. For this purpose, we used the method described by^37^ in a Roche LightCycler® 480 thermocycler provided of the LightCycler® 480 software v1.5.1 (Roche Diagnostics GmbH, Basel, Switzerland). All the samples were analyzed in duplicate. NTC and *N. ceranae* positive control were also included. *N. ceranae* load was expressed as pg/µL. Afterwards, in order to determine whether any change in the microbiota was evident in those bees with the highest level of microsporidian infection, those *N. ceranae*-infected bees with the highest load (> 0.7 pg/µL) were classified in a group as highly infected bees. This threshold was established according to the mean *N. ceranae* load found in 21-day-old worker bees infected in spring in a previous work, which was the day with the significantly highest *N. ceranae* load^38^.

### Metataxonomic studies

#### Sample selection

The selection of samples for the metataxonomic analysis is shown in Table 1. In a first batch of samples (old bees from spring) both AS and PS were processed and analyzed by a metataxonomic approach. After analysing the results of this first analysis (see below), it was decided to continue only with AS and, therefore, the rest of metataxonomic studies were focused only on the AS portions. All the samples were analyzed individually.

#### DNA extraction

The remaining content from the homogenized samples (200 µL in the case of AS and 250 µL in that of PS) were centrifuged for 15 min at 11,000×*g* at 4 °C. DNA extraction from the pellets was performed as described in^39^ using the QIAamp DNA Stool Mini Kit (Qiagen, Hilden, Germany), including a mechanical lysis step with FastPrep Fp120 (Thermo Scientific, Waltham, MA) and glass beads matrix tubes (3 cycles × 60 s, speed 6) in step 4. RNA was removed using ribonuclease A (10 mg/mL) and incubated at 37 °C for 15 min while the protein fraction was removed with proteinase K (10 min at 70 °C). Then, the extracted DNA was eluted in 20 µL of nuclease-free water and its concentration was estimated with a ND-1000 UV spectrophotometer (Nano Drop Technologies, Wilmington, DE). The samples were stored at − 20 °C until further analysis.

#### PCR amplification and sequencing

In order to amplify a fragment of the V3-V4 hypervariable region of the bacterial 16S rRNA gene, a dual-barcoded 2-step PCR reaction was conducted. The amplicons from the V3-V4 hypervariable region were generated using equimolar concentrations of the universal primers S-D-Bact-0341-b-S-17 (ACACTGACGACATGGTTCTACACCTACGGGNGGCWGCAG) and S-D-Bact-0785-a-A-21 (TACGGTAGCAGAGACTTGGTCTGACTACHVGGGTATCTAATCC) as previously described^40^. Primers were synthesised by Isogen Life Sciences (Castelldefels, Spain). To allow for the separation of forward and reverse sequences, Illumina sequencing barcodes used appended to 3’ and 5’ terminal ends of the PCR amplicons. The pooled, purified and barcoded DNA amplicons were sequenced using the Illumina MiSeq 2 × 300 bp paired-end protocol (Illumina Inc., San Diego, CA, USA) following the manufacturer’s recommendations at the facilities of Parque Científico de Madrid (Tres Cantos, Spain). Four negative controls (including one DNA extraction control with PBS and three with nuclease-free water (Sigma®)) instead of a sample, exposed to the same containers, followed the same procedure of the DNA extraction and purification earlier explained to assess possible contaminations. Since there was no amplification detected after the first PCR in any of the blank samples, they were no further sequenced.

After the first PCR, products from the samples were run in agarose gel after being pooled at approximately equimolar DNA concentration. Bands of correct size were excised and purified using QIAEX II Gel extraction Kit (Qiagen) and afterwards quantified with PicoGreen (BMG Labtech, Jena, Germay). Next, a second PCR reaction was carried out and the purified barcoded DNA amplicons were sequenced using Illumina MiSeq pair-end protocol for the construction of libraries (Illumina Inc., San Diego, CA).

Sequences were demultiplexed using the Illumina software (version 2.6.2.3), according to the manufacturer’s guidelines. After the demultiplexing step, the bioinformatics analyses were performed using QIIME 2 2019.1^41^ and the R software (version 3.5.1, https://www.r-project.org/)^42^.

For denoising, DADA2 pipeline^43^ was used following this set: the forward reads were truncated at position 295 and their first 15 nucleotides were trimmed, while the reverse ones were truncated at the position 258 and their first 7 nucleotides were trimmed, to discard positions for which nucleotide median quality was Q19 or below. Taxonomy data was assigned to each amplicon sequence variant (ASV) using the q2-feature-classifier^44^ classify-sklearn naive Bayes taxonomy classifier against the SILVA 138.1 reference database^45^. These taxonomic classifications of 16S-gene amplicon sequences were optimized with the QIIME 2’s q2-feature-classifier^44^

The decontam package version 1.2.1^46^ was used to identify, visualize and remove contaminating DNA with four negative control samples.

#### Statistical and bioinformatics analysis

A table of amplicon sequence variants (ASVs) counts per sample was generated, and bacterial taxa abundances were normalized with the total sum scaling normalization method dividing each ASV count by the total library size in order to yield their relative proportion of counts for each sample. Alpha diversity was studied with the Shannon and Simpson diversity indices with the R vegan package (Version: 2.5.6)^47^. Initially, a first comparison of the microbiota of the bees was carried out to assess whether there were differences depending on the colony from which the samples were taken. Subsequently, the parameters compared in the statistical analysis were the infection by *N. ceranae* (infected vs. non-infected and highly infected vs. non-infected), season period (spring vs. autumn), age of the bee (old vs. young groups), and, in the case of the old group in spring, AS vs. PS and infection status within both groups. Principal coordinates analysis (PCoA) was used in order to evaluate beta diversity and to plot patterns of bacterial community diversity through a distance matrix containing a dissimilarity value for each pairwise sample comparison. Quantitative data were expressed as the median and interquartile range (IQR). Differences between bees’ groups were assessed using Wilcoxon rank sum tests to calculate comparisons between groups with Bonferroni corrections for multiple comparisons. Quantitative (relative abundance) and qualitative (presence/absence) analyses were performed with the Bray–Curtis dissimilarity index and binary Jaccard index, respectively. Analysis of variance of the distance matrices were performed with the “nonparametric MANOVA test” Adonis with 999 permutations (PERMANOVA) as implemented in the R vegan package to reveal statistical significance. The linear discriminant analysis (LDA) effect size (LEfSe) algorithm^48^ was performed with the online interface Galaxy^49^. Heatmaps were performed with the Gplots package 3.1.1 version of the R software. The cladograms were performed with the Hclust hierarchical cluster analysis with complete linkage method from the R’s core package “stats”.

## Results

### Comparison of the AS and PS bacteriomes

After observing that there were no differences among the different recipient colonies in relation to either alpha-diversity (p = 0.47 and p = 0.37 for the Shannon and Simpson diversity indices, respectively) and beta-diversity (p = 0.31 and p = 0.21 for Bray Curtis and binary Jaccard tests, respectively), a comparison between the AS and PS bacteriomes was performed in order to know the best intestinal section to determine the influence of *Nosema* infection in the bees’ bacteriome. A first batch of 81 samples (39 PS and 42 AS samples) from 46 bees from the group of old bees of spring were analyzed. Overall, this analysis rendered 155 different genera, from a number of 2,269,296 high quality sequences. After the alpha-diversity and the PCoA analyses, a strong effect of intestinal section on the microbiome composition was observed. The alpha diversity was significantly higher in the AS samples (Shannon index = 1.5 [1.13–1.65]; Simpson index = 0.69 [0.53–0.75]) than in the PS samples (Shannon index = 1.23 [0.98–1.42]; Simpson index = 0.56 [0.46–0.67]) (p = 0.006 and p = 0.004, respectively). The beta diversity was also significantly different, in terms of relative abundance (Bray–Curtis, p = 0.002) and presence/absence (Binary-Jaccard, p = 0.001) (Suppl. Fig. 1).

When comparing the PS microbiome between infected (n = 21) and non-infected (n = 18) bees, there were no significant differences between both groups neither in the alpha-diversity (Shannon = 1.23 [1.01–1.42] and 1.21 [0.98–1.39], respectively; p = 0.98) nor in the beta-diversity (Bray–Curtis, p = 1.00 and Binary Jaccard, p = 0.66) (Suppl. Fig. 2). The AS bacteriome of infected (n = 21) and non-infected (n = 21) bees was also compared, showing higher alpha diversity in the non-infected samples (Shannon index = 1.57 [1.4–1.68]) than in the infected ones (Shannon index = 1.36 [1.04–1.61]) (p = 0.046). However, there was no impact on beta diversity neither in terms of relative abundance (Bray–Curtis, p = 0.26) nor in presence/absence (Binary-Jaccard, p = 0.3) (Suppl. Fig. 3).

### Description of the AS microbiome of bees: influence of *Nosema* infection

As the main differences between infected and non-infected bees were found in AS samples, this gut section was selected for the next analysis, now including all the available samples (n = 158). The metataxonomic analysis of the AS samples yielded 11,280,822 high quality reads, ranging from 18,761 to 121,423 reads per sample [median (IQR) = 72,679 (56,997–86,611)], which corresponded to 440 different ASVs. Overall, a total of 19 phyla and 238 genera were identified. The most abundant phyla were Proteobacteria, Firmicutes, Actinobacteriota, Bacteroidota and Acidobacteriota.

**Figure 2:**
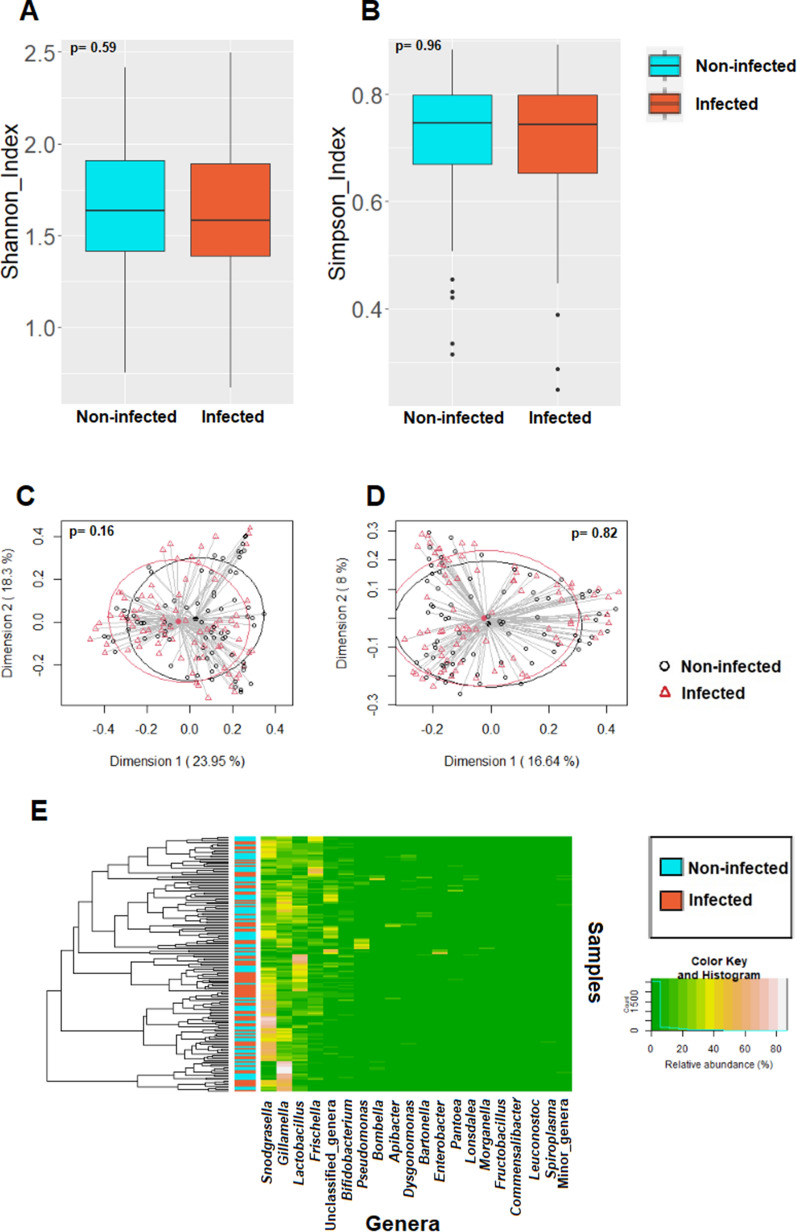
Comparison between the values of alpha and beta diversity and statistical analysis (Wilcoxon rank test and PERMANOVA test, respectively), at the ASV level, of the 158 AS samples grouped by *Nosema* spp. infection. (**A**) Shannon diversity index; (**B**) Simpson diversity index ; (**C**) PCoA plots based on the Bray–Curtis dissimilarity index; (**D**) PCoA plots based on the Jaccard’s coefficient for binary data (presence of absence). (**E**) Heatmap plot representing the hierarchical clustering (hclust with the complete linkage method for hierarchical clustering), at the genus level, of the AS samples by *Nosema* infection cohorts.

Alpha diversity of the AS samples was analyzed in the group of infected bees (n = 80; Shannon index = 1.58 [1.39–1.89]; Simpson index = 0.74 [0.65–0.80]) and in the group of non-infected bees (n = 78; Shannon index = 1.64 [1.41–1.91]; Simpson index = 0.75 [0.67–0.80]) and no significant differences were found (p = 0.59 and p = 0.96, respectively). Beta diversity was also analyzed and compared between both groups and no statistically significant differences were found neither in terms of relative abundance (Bray–Curtis, p = 0.16) nor in terms of presence/absence (Binary-Jaccard, p = 0.82). In addition, no significant differences were detected in the relative abundances of the main phyla and genera (Fig. 2; Table 2).

**Table 2:** Relative frequencies, medians and interquartile ranges (IQR) of the most abundant bacterial phyla (bold) and genera (italics) detected in AS of non-infected and infected bees.

Phylum/genera	Non-infected		Infected		p-value^†^
	n (%)*	Median (IQR)	n (%)*	Median (IQR)	
**Proteobacteria**	78 (100%)	81.41 (71.05–90.23)	80 (100%)	78.8 (66.6–88.16)	0.51
*Snodgrassella*	78 (100%)	24.09 (10.53–39.73)	80 (100%)	33.64 (16.71–44.38)	0.1
*Gilliamella*	78 (100%)	21.2 (14.99–35.6)	80 (100%)	20.31 (12.64–29.36)	0.18
*Frischella*	75 (96.15%)	6.17 (0.32–13.89)	73 (91.25%)	5.43 (0.44–12.27)	0.46
*Pseudomonas*	39 (50%)	< 0.01 (< 0.01–0.06)	33 (41.25%)	< 0.01 (< 0.01–0.05)	0.39
*Bombella*	50 (64.1%)	0.02 (< 0.01–0.11)	50 (62.5%)	0.04 (< 0.01–0.21)	0.62
*Bartonella*	22 (28.21%)	< 0.01 (< 0.01–0.01)	19 (23.75%)	< 0.01 (< 0.01–< 0.01)	0.48
*Enterobacter*	15 (19.23%)	< 0.01 (< 0.01–< 0.01)	13 (16.25%)	< 0.01 (< 0.01–< 0.01)	0.71
*Pantoea*	16 (20.51%)	< 0.01 (< 0.01–< 0.01)	15 (18.75%)	< 0.01 (< 0.01–< 0.01)	0.73
*Lonsdalea*	17 (21.79%)	< 0.01 (< 0.01–< 0.01)	20 (25%)	< 0.01 (< 0.01–< 0.01)	0.75
*Morganella*	14 (17.95%)	< 0.01 (< 0.01–< 0.01)	13 (16.25%)	< 0.01 (< 0.01–< 0.01)	0.83
*Commensalibacter*	27 (34.62%)	< 0.01 (< 0.01–0.02)	26 (32.5%)	< 0.01 (< 0.01–0.02)	0.69
**Firmicutes**	78 (100%)	15.61 (7.68–21.98)	80 (100%)	16.07 (8.91–24.58)	0.62
*Lactobacillus*	78 (100%)	13.34 (7.41–21.97)	80 (100%)	15.4 (8.74–24.38)	0.67
*Fructobacillus*	26 (33.33%)	< 0.01 (< 0.01–0.02)	19 (23.75%)	< 0.01 (< 0.01–< 0.01)	0.38
*Leuconostoc*	14 (17.95%)	< 0.01 (< 0.01–< 0.01)	16 (20%)	< 0.01 (< 0.01–< 0.01)	0.9
*Spiroplasma*	0 (0%)	< 0.01 (< 0.01–< 0.01)	2 (2.5%)	< 0.01 (< 0.01–< 0.01)	0.16
**Actinobacteriota**	76 (97.44%)	2.27 (0.76–4.62)	76 (95%)	1.74 (0.43–6.27)	0.56
*Bifidobacterium*	75 (96.15%)	2.19 (0.66–4.51)	74 (92.5%)	1.69 (0.43–6.27)	0.64
**Bacteroidota**	61 (78.21%)	0.04 (< 0.01–0.26)	56 (70%)	0.04 (< 0.01–0.33)	0.66
*Apibacter*	16 (20.51%)	< 0.01 (< 0.01–< 0.01)	21 (26.25%)	< 0.01 (< 0.01–< 0.01)	0.5
*Dysgonomonas*	15 (19.23%)	< 0.01 (< 0.01–< 0.01)	11 (13.75%)	< 0.01 (< 0.01–< 0.01)	0.33
**Acidobacteriota**	13 (16.67%)	< 0.01 (< 0.01–< 0.01)	13 (16.25%)	< 0.01 (< 0.01–< 0.01)	0.86
**Minor_phyla**	29 (37.18%)	< 0.01 (< 0.01–0.01)	26 (32.5%)	< 0.01 (< 0.01–< 0.01)	0.74
Minor_genera	72 (92.31%)	0.14 (0.03–0.52)	71 (88.75%)	0.14 (0.02–0.3)	0.8
Unclassified_genera	78 (100%)	1.88 (0.55–6.6)	80 (100%)	2.8 (0.32–9.42)	0.62

*Number of samples in which the phylum/genus was detected (relative frequency of detection).

^†^Wilcoxon rank sum test with Bonferroni correction.

Subsequently, the AS bacteriome of the group of highly infected bees (bees with *N. ceranae-PTP3* load > 0.7 pg/µL; n = 12) was compared to the AS bacteriome of non-infected bees (n = 78). Again, there were no differences in the alpha diversity between highly infected (Shannon index = 1.40 [1.20–1.70]; Simpson index = 0.66 [0.60–0.78]) and non-infected samples (Shannon index = 1.64 [1.41–1.91]; Simpson index = 0.75 [0.67–0.80]) (p = 0.068 and p = 0.18, respectively). There were no differences in beta diversity in terms of relative abundance (Bray–Curtis, p = 0.71) but there were differences regarding the presence/absence (Binary-Jaccard, p = 0.026). At the taxonomic level, differences were found in relation to the phylum Proteobacteria, being its relative abundance higher in highly infected bees (89.01% [84.6–96.36]) than in non-infected bees (81.41% [71.05–90.23]) (p = 0.018), and to the phylum Firmicutes, which abundance was higher in non-infected bees (15.61% [7.68–21.98]) than in highly infected ones (8.51% [3.13–10.76]) (p = 0.003). Genus *Lactobacillus* had higher relative abundance in non-infected (13.34% [7.41–21.97]) than in highly infected bees (7.29% [3.1–10.75]) (p = 0.003), and the same was observed for the genus *Bartonella* which relative abundance was also higher in non-infected bees (p = 0.038).

### Influence of the season on the AS bacteriome

**Figure 3:**
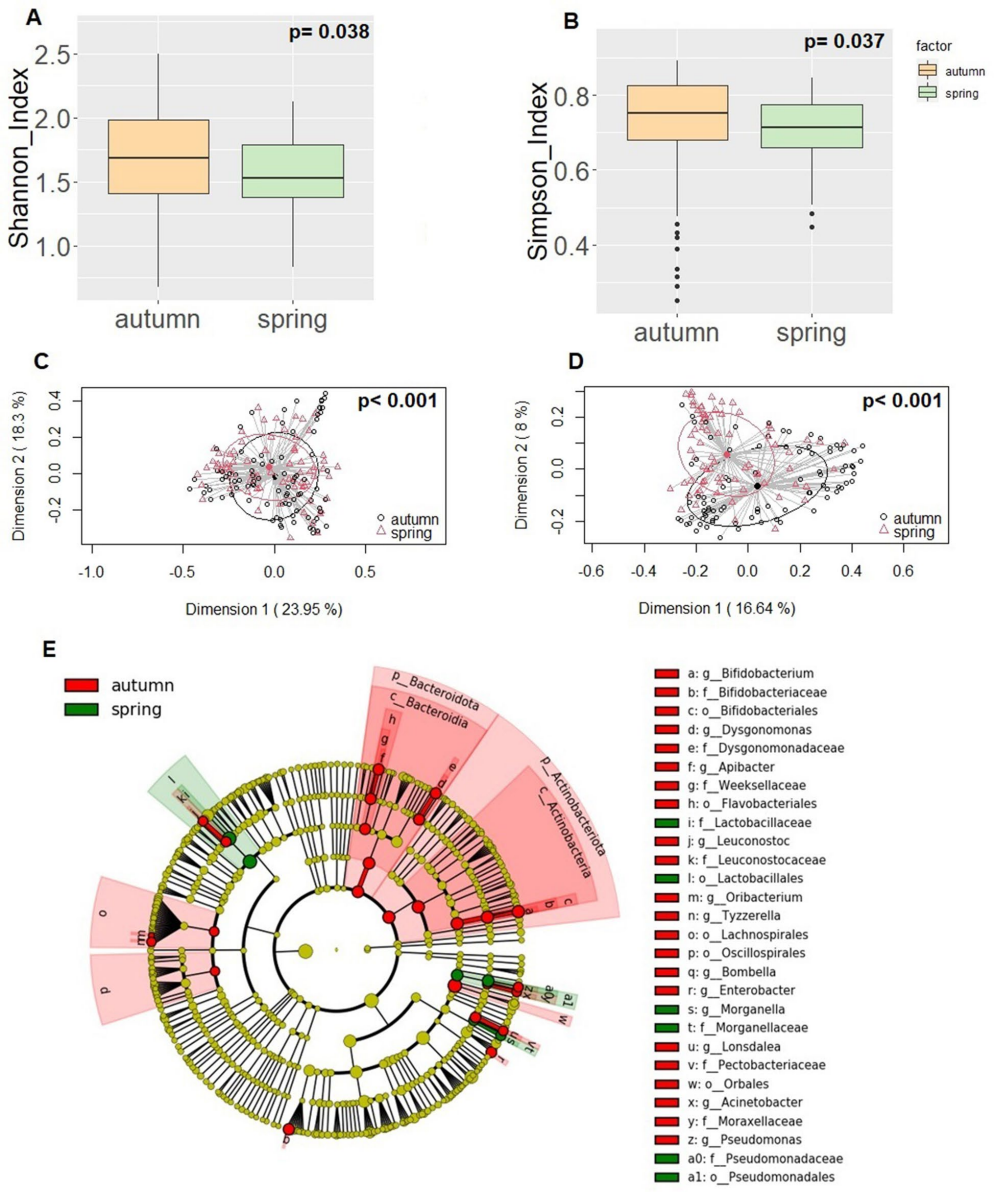
Comparison between the values of alpha and beta diversity and statistical analysis (Wilcoxon rank test and PERMANOVA test, respectively), at the ASV level, of the 158 AS samples grouped by the beekeeping season period. (**A**) Shannon diversity index; (**B**) Simpson diversity index ; (**C**) PCoA plots based on the Bray–Curtis dissimilarity index; (**D**) PCoA plots based on the Jaccard’s coefficient for binary data (presence of absence). (**E**) LEfSe analysis identifying taxonomic differences in the microbiota of samples grouped by the beekeeping season period. Differentially abundant bacterial taxa were identified using linear discriminant analysis (LDA) and the effect size (LEfSe) algorithm. Cladogram showing the LEfSe comparison of differential bacterial taxa. The central point represents the root of the bacterial tree and each ring the next lower taxonomic level from phylum to genus (from the inner to the outer ring: phylum, class, order, family, and genus). The color node (other than yellow) indicates which taxa are significantly higher in relative abundance.

Spring and autumn seasons were also studied to determine their effect on the honeybees’ microbiome. Initially, the effect of spring (n = 71) *vs.* autumn (n = 87) on the diversity and composition of the bees´ bacteriome was assessed regardless the infection status. Alpha diversity in the spring group (Shannon index = 1.53 [1.38–1.79]; Simpson index = 0.71 [0.66–0.78]) was significantly lower than that observed in the autumn group (Shannon index = 1.69 [1.41–1.98]; Simpson index = 0.75 [0.68–0.83]; p = 0.038 and p = 0.037, respectively). The season factor also exerted a strong impact on beta diversity, both in terms of relative abundance (Bray-Courtis, p < 0.001) and presence/absence (Binary-Jaccard p < 0.001) (Fig. 3).

Relevant differences in the composition at the phyla level were observed for Proteobacteria, Firmicutes, Actinobacteriota, Bacteroidota and Acidobacteriota (Table 3). Proteobacteria was the most abundant phylum in both groups, although it was more abundant in spring (p < 0.001) (Table 3). On the contrary, the relative abundances of Firmicutes, Actinobacteriota and Bacteroidota were higher in the autumn group (p = 0.044, p < 0.001 and p < 0.001, respectively) (Table 3).

**Table 3:** Relative frequencies, medians and interquartile range (IQR) of the most abundant bacterial phyla (bold) and genera (italics) detected in the AS of bees collected in autumn and spring.

Phylum/Genera	Autumn		Spring		p-value^†^
	n (%)*	Median (IQR)	n (%)*	Median (IQR)	
**Proteobacteria**	87 (100%)	75.84 (64.78–85.17)	71 (100%)	83.7 (75.23–92.37)	< 0.001
*Snodgrassella*	87 (100%)	23.68 (10.13–39.85)	71 (100%)	35.32 (20.31–49.68)	0.004
*Gilliamella*	87 (100%)	18.51 (11.82–29.19)	71 (100%)	22.49 (15.41–33.94)	0.076
*Frischella*	86 (99%)	7.13 (0.63–13.72)	62 (87%)	4.29 (0.18–12.29)	0.100
*Pseudomonas*	49 (56%)	0.01 (< 0.01–0.07)	23 (32%)	< 0.01 (< 0.01–0.04)	0.040
*Bartonella*	26 (30%)	< 0.01 (< 0.01–0.03)	15 (21%)	< 0.01 (< 0.01–< 0.01)	0.130
*Commensalibacter*	40 (46%)	< 0.01 (< 0.01–0.05)	13 (18%)	< 0.01 (< 0.01–< 0.01)	< 0.001
*Bombella*	73 (84%)	0.08 (0.02–0.33)	27 (38%)	< 0.01 (< 0.01–0.05)	< 0.001
*Enterobacter*	21 (24%)	< 0.01 (< 0.01–< 0.01)	7 (10%)	< 0.01 (< 0.01–< 0.01)	0.025
*Pantoea*	19 (22%)	< 0.01 (< 0.01–< 0.01)	12 (17%)	< 0.01 (< 0.01–< 0.01)	0.600
*Lonsdalea*	37 (43%)	< 0.01 (< 0.01–0.06)	0 (0%)	< 0.01 (< 0.01–< 0.01)	< 0.001
*Morganella*	7 (8%)	< 0.01 (< 0.01–< 0.01)	20 (28%)	< 0.01 (< 0.01–0.03)	< 0.001
**Firmicutes**	87 (100%)	17.11 (10.46–25.25)	71 (100%)	13.4 (6.02–21.87)	0.044
*Lactobacillus*	87 (100%)	16.05 (10.36–24.70)	71 (100%)	12.24 (5.91–20.2)	0.054
*Fructobacillus*	21 (24%)	< 0.01 (< 0.01–< 0.01)	24 (34%)	< 0.01 (< 0.01–0.03)	0.100
*Leuconostoc*	28 (32%)	< 0.01 (< 0.01–0.02)	2 (3%)	< 0.01 (< 0.01–< 0.01)	< 0.001
*Spiroplasma*	1 (1.15%)	< 0.01 (< 0.01–< 0.01)	1 (1%)	< 0.01 (< 0.01–< 0.01)	0.890
**Actinobacteriota**	86 (99%)	4.38 (1.88–7.30)	66 (93%)	0.93 (0.31–1.9)	< 0.001
*Bifidobacterium*	85 (98%)	4.38 (1.84–7.30)	64 (90.14%)	0.87 (0.31–1.89)	< 0.001
**Bacteroidota**	74 (85%)	0.06 (0.01–0.65)	43 (60%)	0.01 (< 0.01–0.06)	< 0.001
*Dysgonomonas*	21 (24%)	< 0.01 (< 0.01–< 0.01)	5 (7.04%)	< 0.01 (< 0.01–< 0.01)	0.005
*Apibacter*	27 (31%)	< 0.01 (< 0.01–0.04)	10 (14.08%)	< 0.01 (< 0.01–< 0.01)	0.014
**Acidobacteriota**	22 (25%)	< 0.01 (< 0.01–< 0.01)	4 (6%)	< 0.01 (< 0.01–< 0.01)	< 0.001
**Minor_phyla**	31 (36%)	< 0.01 (< 0.01–< 0.01)	24 (34%)	< 0.01 (< 0.01–0.01)	0.980
Minor_genera	74 (85%)	0.15 (0.01–0.55)	69 (97%)	0.14 (0.05–0.28)	0.440
Unclassified_genera	87 (100%)	2.12 (0.62–8.70)	71 (100%)	2.36 (0.24–7.48)	0.850

*Number of samples in which the phylum/genus was detected (relative frequency of detection).

^†^Wilcoxon rank sum test with Bonferroni correction.

At the genus level, significant differences were also observed between both groups (Table 3). *Snodgrassella* was the most abundant genus in both groups but was more abundant in the spring group (35.32% [20.31–49.68]), than in the autumn one (23.68% [10.13–39.85]) (p = 0.004). On the contrary, the genera *Leuconostoc*, *Bifidobacterium*, *Dysgonomonas* and *Apibacter* were more abundant among autumn bees. Other major genera, including *Gilliamella, Frischella* and *Lactobacillus*, showed a similar proportion in both groups (Table 3).

The LEfSe comparison between the spring and autumn groups corroborated most of the results cited above; the autumn group bacteriome was characterized by a predominance of the phyla Actinobacteriota, mainly because of the strong influence of the order Bifidobacteriales (genus *Bifidobacterium*), and Bacteroidota, mainly because of the strong influence of the families Dysgomonadaceae (genus *Dysgonomonas*) and Weeksellaceae (genus *Apibacter*), among other taxa. However, it must be highlighted that *Dysgonomonas* and *Apibacter* had a very low abundance (< 0.01) and are usually rare in the bee’s microbiome. The spring group bacteriome was characterized by a more subtle predominance of specific taxa, including families Lactobacillaceae, Pseudomonadaceae and Morganellaceae, and the genus *Morganella*, as assessed by the LEfSe approach (Fig. 3E).

Lastly, the influence of the *Nosema* infection status within each seasonal group was assessed. No differences in either alpha or beta diversity were observed between non-infected and infected bees neither in spring nor in autumn (Suppl. Figs. 4 and 5). Statistical differences among the most abundant phyla were only found in spring for the phylum Actinobacteriota, being its relative abundance of 1.06% (0.55–2.43) and 0.67% (0.07–1.27) in non-infected and infected bees, respectively (p = 0.04) (Suppl. Table 1), and for the genus *Bifidobacterium* in non-infected and infected bees (1.06 [0.56–2.38] and 0.63 [0.04–1.25], respectively) bees (p = 0.041). No statistical differences among the most abundant phyla nor more abundant genera were found in autumn samples (Suppl. Table 2).

### Influence of age on the AS bacteriome

The age of the bees was also studied to determine its role in the shaping of the bees’ microbiome. Two different groups, younger (n = 58) and older (n = 100) bees, were analyzed firstly without taking into account the infection status and, later, including the *N. ceranae* infection factor in the comparison.

In the first case, age-related differences were observed in relation to alpha diversity indices (Shannon index = 1.54 [1.3–1.72]; Simpson index = 0.72 [0.64–0.77] in the younger group; Shannon index = 1.69 [1.43–1.93]; Simpson index = 0.75 [0.68–0.81] in the older one) (p = 0.054; p = 0.038 respectively), and, also, to beta diversity indices (Bray-Courtis, p < 0.001; Binary-Jaccard, p < 0.001). Statistical differences among the most abundant phyla were only found for the phylum Actinobacteriota, being its relative abundance of 1.56% [0.09–3.23] and 2.25% [0.89–6.21] in younger and older bees, respectively (p = 0.03) and the phylum Bacteroidota 0.02% [< 0.01–0.06] and 0.06% [< 0.01–0.92] in younger and older bees, respectively (p = 0.03). At the genus level, significant differences were also observed between both groups: *Snodgrassella* was, on average, the most abundant genus and it was more abundant in the younger group (38.17% [21.71–51.12]), than in the older group (22.88% [11.15–41.02] of the sequences) (p = 0.002). In addition, significant differences were also observed between both groups in the *Gilliamella* relative abundance; this genus was more abundant in the older group 25.26 [16.57–36.65], than in the younger group (14.75% [7.09–21.73] of the sequences) (p < 0.001) (Supp. Table S3).

Regarding the influence of the *N. ceranae* infection status within each age group, no differences in either alpha or beta diversity were observed between non-infected and infected bees, neither among young nor among old bees. Statistical differences among the most abundant phyla were only found in the young group for the phylum Proteobacteria, being its relative abundance of 82.04% [75.26–91.8] and 75.46% [64.33–80.77] in non-infected and infected bees, respectively (p = 0.02), and for the genus *Apibacter*, which abundance was higher in the infected group (p = 0.049).

## Discussion

The aim of the study was to determine whether *N. ceranae* infection modifies the bacteriome of honeybees in order to understand the interactions between the parasite and its niche. Our results show that this infection only produces slight modulations on the bees’ microbiome as no differences in alpha or beta diversity between infected and non-infected honeybees were observed.

In order to know the best intestinal section to determine the influence of *Nosema* infection in the microbiome, a first batch of 81 samples (42 AS and 39 PS samples) were analyzed. Although the high number of bacteria in the ileum could influence the results of the AS, our main objective was to study the environment of infection and the areas closest to it. Therefore, we divided the intestine into two sections (AS and PS), trying to minimize the bias of studying the entire digestive tract and avoid possible errors associated with inaccurate dissection. In fact, a strong effect of intestinal section on the microbiome composition was observed, since AS had higher alpha and beta diversities. Differences in microbiome composition according to the anatomic part of the gut are supported by the literature^3,6,8^, with a lower abundance of bacteria in midgut probably due to the presence of the peritrophic membrane and a variety of digestive enzymes^8^. In this first batch of samples, significant differences were only observed within the AS group when comparing the bacteriome between infected bees and non-infected bees; in fact, the infection by *N. ceranae* seemed to reduce the diversity of bacteria in the surrounding environment of the infection. That was the rationale why the subsequent analyses were exclusively focused on AS samples.

However, when the 158 bees were analysed (80 infected and 78 non-infected), no statistical differences were found neither in the alpha diversity nor in the beta diversity in terms of relative abundance and presence/absence of genus. When only the highest infected bees were compared to the non-infected group, only a few differences were found. Therefore, despite the fact that the life cycle of *N. ceranae* develops within the epithelial cells of the ventriculus and the extensive damage in the epithelium caused by the microsporidia^20,21,50,51^, this seems not be highly reflected into the bacteriome composition of the surrounding environment of the infection. This is in accordance to previous studies done on bees kept under colony conditions in which only a subtle effect of this infection in the bacteriome composition was also reported^52,53^. However, this issue remains controversial since, on the contrary, other studies with bees kept under laboratory conditions showed significant differences in the alpha diversity between control and experimentally infected honeybees^34,54^.

Honeybees feed on nectar, honey and pollen, which must be processed by digestive enzymes to breakdown for further use as a source of carbon and nitrogen. The honeybees’ ventriculi participate in the peritrophic membranes production, nutrient absorption and transport, and enzyme secretion. In fact, microapocrine, holocrine and merocrine secretions are produced in this tissue where lysosomal hydrolases (acid phosphatase and nonspecific esterase) and alkaline phosphatase activity have been identified, which contribute to the digestive process of the food that bees ingest^55,56^. As the *N. ceranae* infection affects this tissue, all these digestive processes are affected and finally reflected in the immunomodulatory effects and metabolism alterations reported in bees infected^24,29^, which eventually lead to shorten their life-span. Therefore, the food ingested by *N. ceranae* infected bees could arrive at least partially processed to the last parts of the gut and this could explain the differences in the relative abundance of some groups of bacteria depending on the infection status. On the other hand, the main number of bacterial communities are located in the ileum and rectum where they digest and absorb nutrients from bee food^57,58^. At these locations, fermentative (*Gilliamella apicola*, *Bifidobacterium* spp. and *Lactobacillus* Firm-4 and Firm-5) and oxidative (*Snodgrasella alvi*) core bacteria have been reported to contribute digesting the food by breaking down the plant polysaccharides present in pollen. This process probably provides the bees access to the products of bacterial-assisted carbohydrate breakdown^3^. Thus, the apparent resilience of the rectum bacteriome after the *N. ceranae* infection might compensate, somehow, the lack of food processing by the infected ventriculus.

Further analyses were performed to assess the role of other variables in the bacteriome composition of the bee’s gut. It has been reported that bee castes, age of the individual and colony modify gut microbial communities, probably due to different host physiology, diet and environment, which could shape the composition of the microbiome^3^. However, in this study no significant differences were found between the recipient colonies used. In fact, seasonality was the variable that most affected bacteriome composition, which is in agreement with previous studies^13–15,59^. Under our experimental conditions, alpha diversity in June was significantly lower than in October, as previously reported^15^. In contrast, other studies have found that the bacteriome is relatively stable throughout June to October^13^, or that there is a higher alpha diversity during beekeeping season than in autumn or winter^14,60^. The bacteriome was, overall, dominated by the phyla Proteobacteria and Firmicutes as previously found for samples collected at the same location^61^. Within Proteobacteria, *Snodgrasella* was the most abundant genus and its relative abundance was higher in spring. On the contrary, other studies have found that the abundance of this genus was higher in autumn than in spring^13,15,59^, and have hypothesized that its presence would protect the honeybees’ gut against potential pathogens that could be accumulated in their bodies until excretion in spring^15^. In this context, we only found statistical differences when the bacteriome composition from *N. ceranae*-infected and non-infected bees collected in spring was compared, with higher relative abundances of the phylum Actinobacteriota and the genus *Bifidobacterium* in non-infected bees.

In relation to the phylum Firmicutes, the abundance of the genus *Lactobacillus* was similar in both seasons. Some species of this genus, which has been reclassified recently^62^, have antimicrobial properties that can inhibit the growth and colonization of potential pathogens and, as a consequence, its presence seems particularly relevant for bees’ health^3,63,64^. This might explain the higher relative abundance of this phylum in the non-infected bees compared to those with the highest parasitic load from the results.

The last studied factor was age. As happened for season, age-related differences were observed in relation to both alpha and beta diversity indices. Microbial communities present in diverse types of worker bees (nurses, foragers and winter bees) are different from each other^14^, which may reflect the influence of the ontogenetic state of the honeybee on the gut microbial composition^9–12,65^. Also, some studies have found differences in the microbial composition of the ventriculi depending on the in-hive tasks^11^, as the honeybee polyethism is an age-related factor. In our study, sequences belonging to the phyla Actinobacteriota and Bacteroidota were more abundant in older bees. Actinobacteriota has been detected in forager crops^12^ and it may be acquired by old interior bees through close contact with foragers. On the other hand, the higher abundance of *Snodgrasella* was higher in young bees than in old ones; this is in accordance to previous reports showing that this genus is mainly acquired through contact with nurses^7^, which in fact are young bees.

Changes in the bacteriome of the honeybees that are infected by pathogens may reflect a dysbiosis state in their guts^18,66,67^, which may be reverse by the use of bee-specific probiotics^64,68^. However, in our study seasonality was the variable that most affected bacteriome composition of the honeybee guts. The paramount relevance of the season as a driver of physiological changes in the bee bacteriome has been highlighted previously^32^. In fact, season and season-associated-food availability that takes place during foraging season and, also, before and after the overwintering period, are major factors explaining natural shifts in the gut bacteriome composition of honeybees^13–15^. On the other hand, although no major changes have been found in the bacteriome composition of *N. ceranae*-infected bees, some studies have shown that the addition of some probiotics can modulate microsporidia infection by reducing the spore counts^64,68–70^ and even reducing the mortality associated^69,70^. Similarly, some prebiotics have also shown to reduce mortality in infected bees even when the level of infection is not reduced^35^. Therefore, this could be a promising future avenue to reduce the consequences of the infection by this pathogen. Further research is therefore needed to elucidate possible associations between the microbiome, nutrition and natural infection by *N. ceranae*, and also, to find efficient, safe and environmentally friendly strategies for prevention and treatment of *N. ceranae* infections through the modulation of the bees’ microbiome.

## Supplementary Information

Supplementary Information.

## Data Availability

The raw microbiome sequencing data are available from NCBI’s Sequence Read Archive under accession no. PRJNA816533.
