# Supplementary material for: Effect of Nosema ceranae infection and season on the gut bacteriome composition of the European honeybee (Apis mellifera)
Source: Sci Rep. 2022 Jun 4;12:9326. doi: 10.1038/s41598-022-13337-4 (PMC9167302; doi:10.1038/s41598-022-13337-4)
Supplement: Supplementary file 1 — Supplementary Information. [file 41598_2022_13337_MOESM1_ESM.pdf]

Supplementary Figure 1. Comparison between the values of alpha and beta diversity and statistical analysis (Wilcoxon rank test and PERMANOVA test, respectively), at the ASV level, of the PS and AS samples. (A) Shannon diversity index; (B) Simpson diversity index ; (C) PCoA plots based on the Bray-Curtis dissimilarity index; (D) PCoA plots based on the Jaccard's coefficient for binary data (presence of absence).

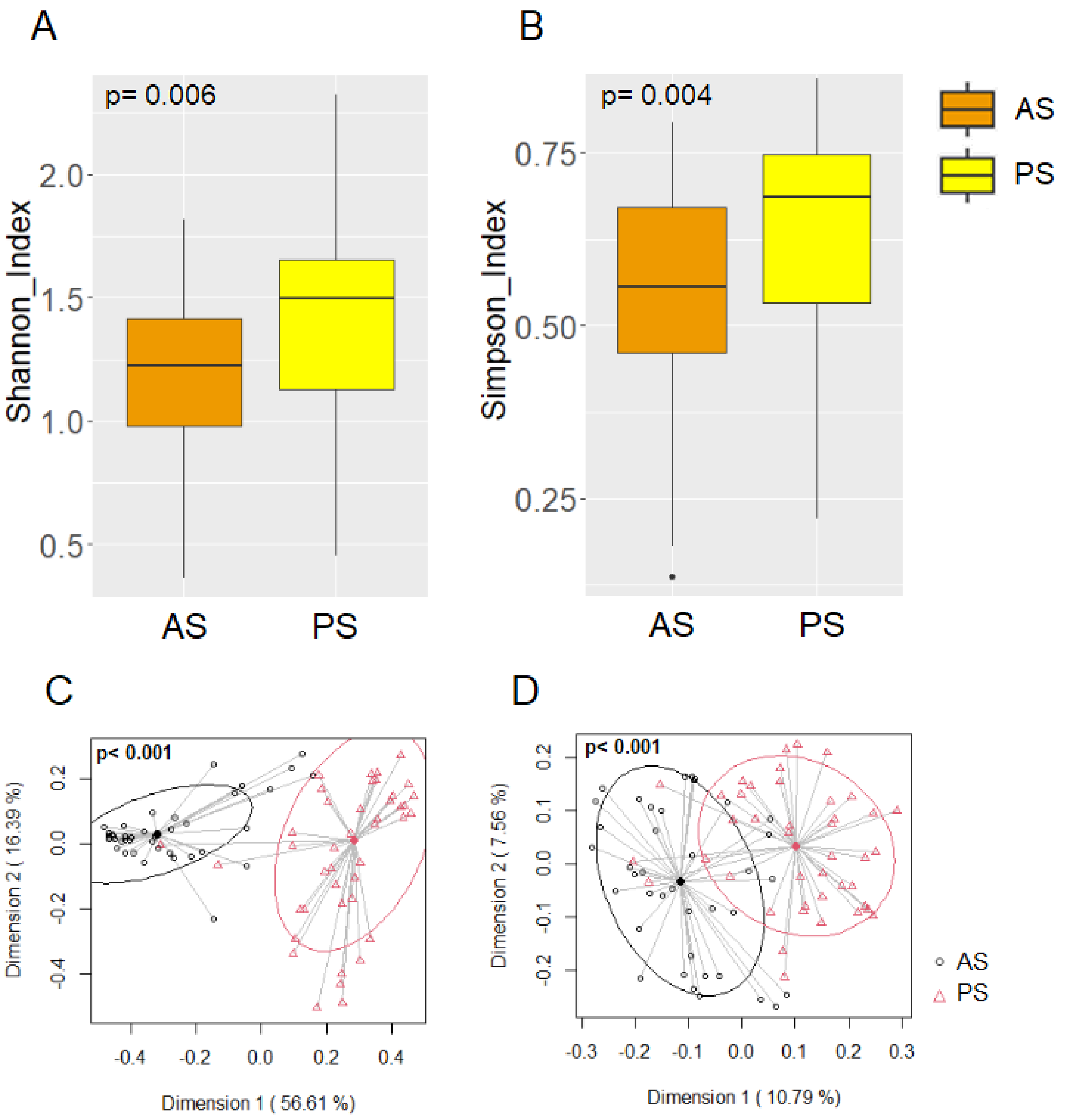

Supplementary Figure 2. Comparison between the values of alpha and beta diversity and statistical analysis (Wilcoxon rank test and PERMANOVA test, respectively), at the ASV level, of the PS samples grouped by *Nosema* spp. infection. (A) Shannon diversity index; (B) Simpson diversity index ; (C) PCoA plots based on the Bray-Curtis dissimilarity index; (D) PCoA plots based on the Jaccard's coefficient for binary data (presence of absence).

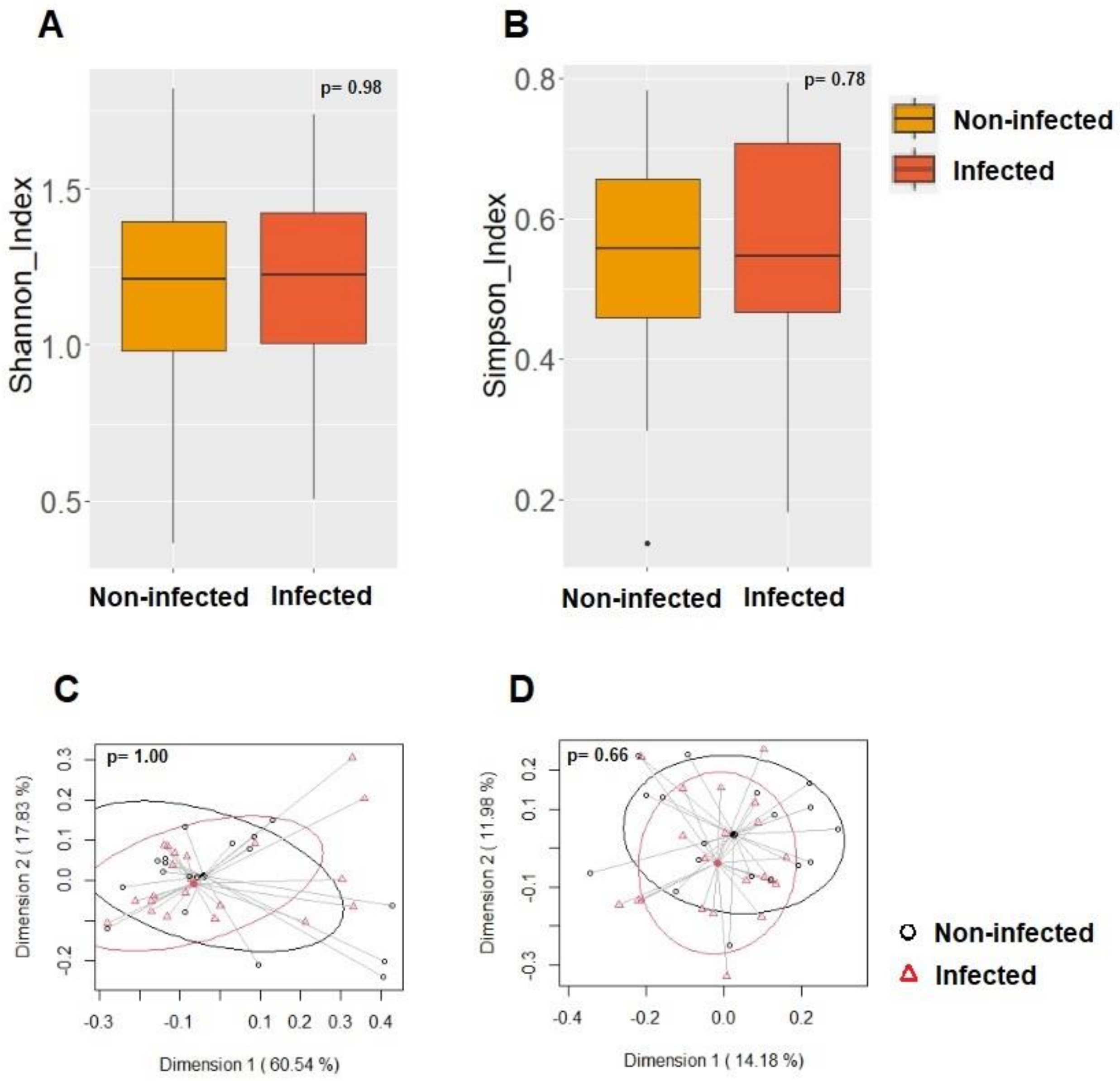

Supplementary Figure 3. Comparison between the values of alpha and beta diversity and statistical analysis (Wilcoxon rank test and PERMANOVA test, respectively), at the ASV level, of the AS samples grouped by *Nosema* spp. infection. (A) Shannon diversity index; (B) Simpson diversity index ; (C) PCoA plots based on the Bray-Curtis dissimilarity index; (D) PCoA plots based on the Jaccard's coefficient for binary data (presence of absence).

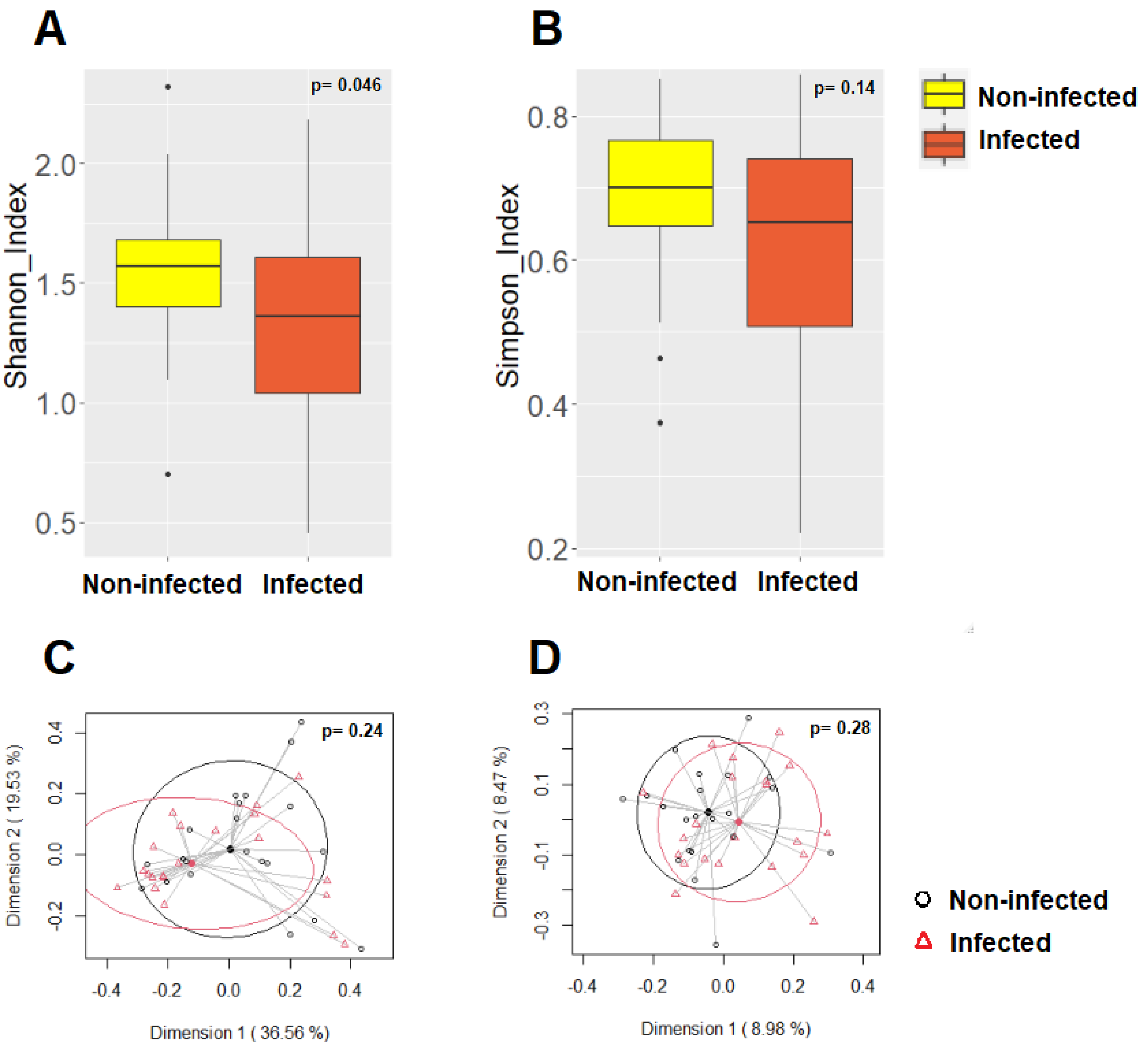

Supplementary Figure 4. Comparison between the values of alpha and beta diversity and statistical analysis (Wilcoxon rank test and PERMANOVA test, respectively), at the ASV level, of the 71 AS samples of the spring period grouped by *Nosema* spp. infection. (A) Shannon diversity index; (B) Simpson diversity index ; (C) PCoA plots based on the Bray-Curtis dissimilarity index; (D) PCoA plots based on the Jaccard's coefficient for binary data (presence of absence). (E) Heatmap plot representing the hierarchical clustering (hclust with the complete linkage method for hierarchical clustering), at the genus level, of the AS samples by *Nosema* infection cohorts.

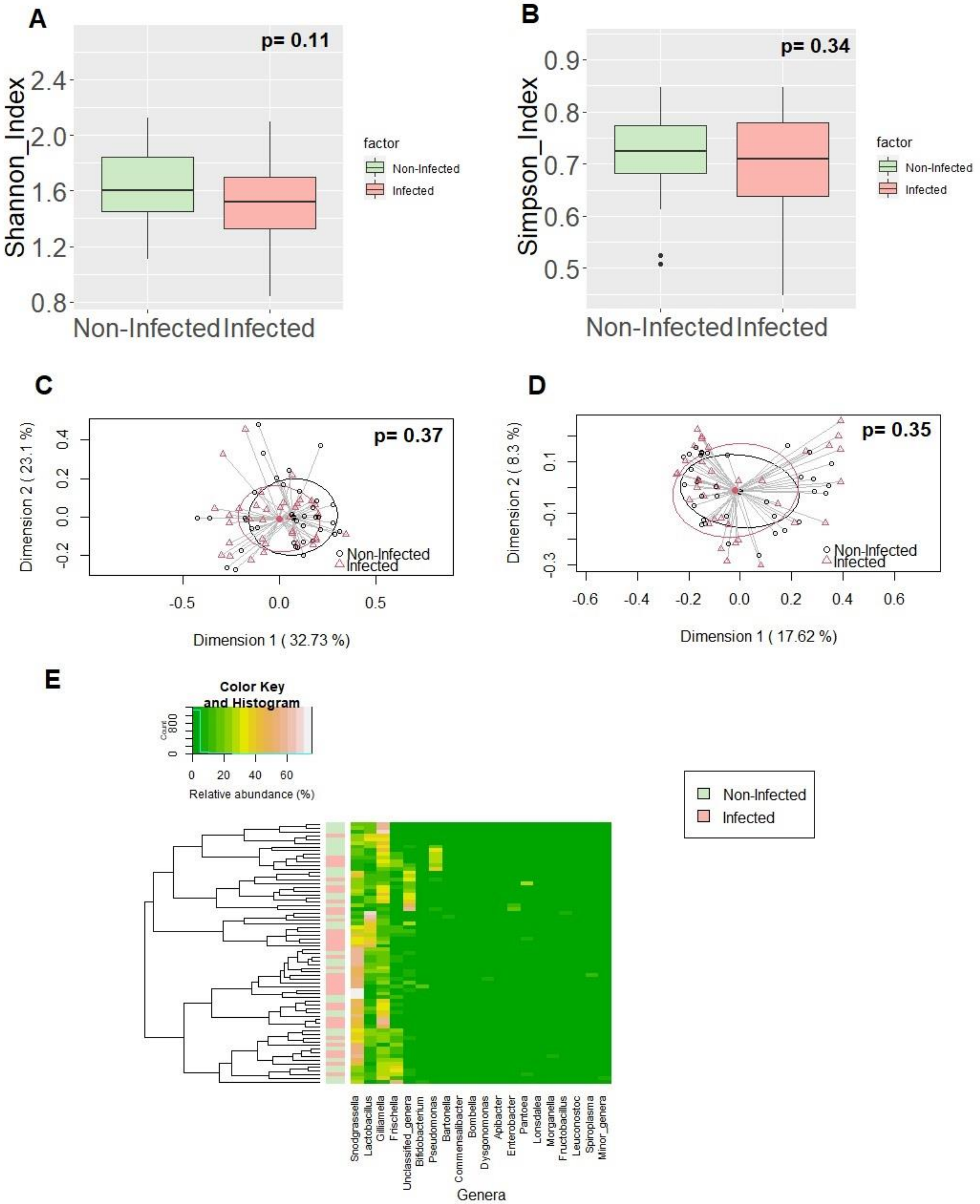

Supplementary Figure 5. Comparison between the values of alpha and beta diversity and statistical analysis (Wilcoxon rank test and PERMANOVA test, respectively), at the ASV level, of the 87 AS samples of the autumn period grouped by *Nosema* spp. infection. (A) Shannon diversity index; (B) Simpson diversity index ; (C) PCoA plots based on the Bray-Curtis dissimilarity index; (D) PCoA plots based on the Jaccard's coefficient for binary data (presence of absence). (E) Heatmap plot representing the hierarchical clustering (hclust with the complete linkage method for hierarchical clustering), at the genus level, of the AS samples by *Nosema* infection cohorts.

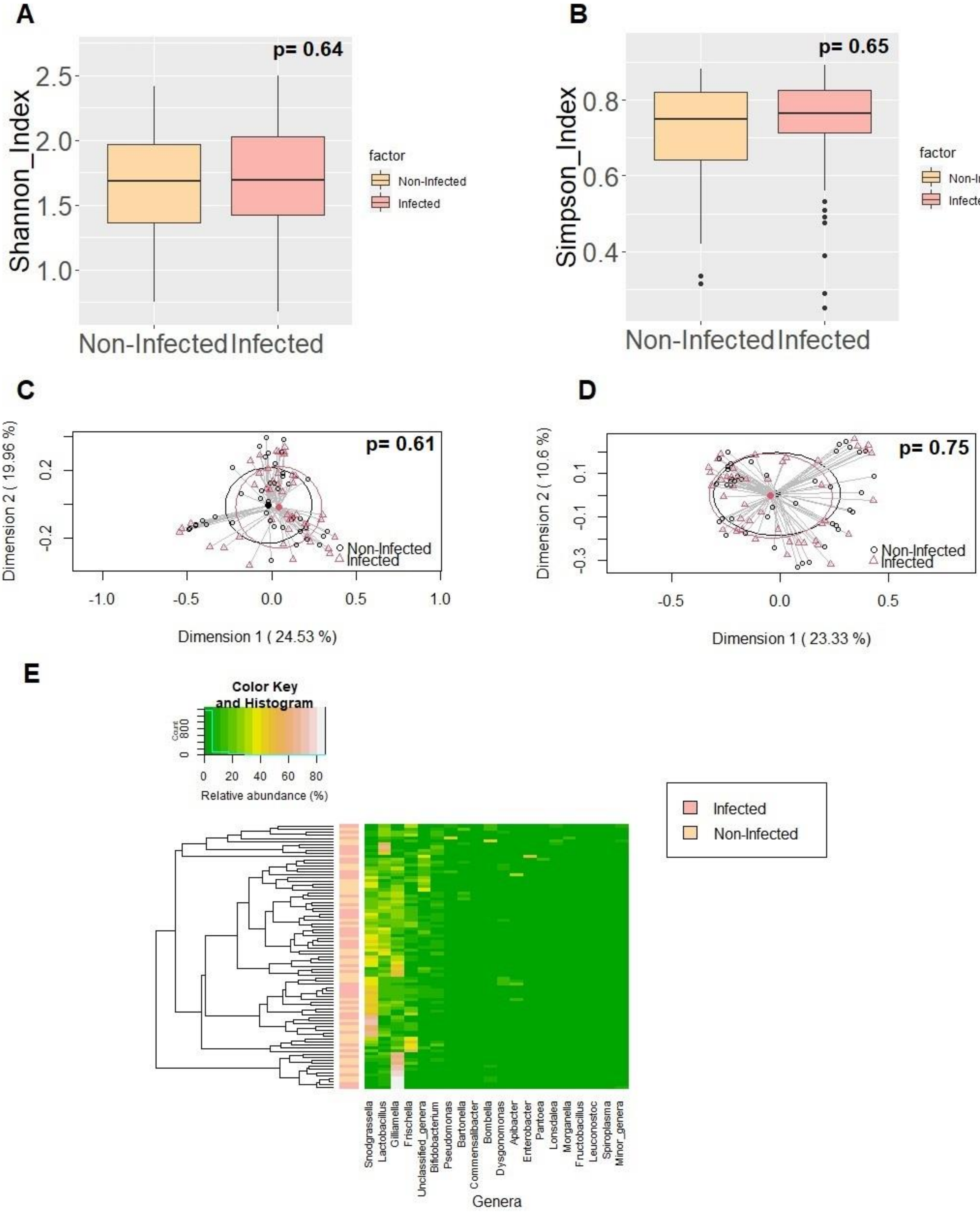

Supplementary Table 1. Relative frequencies, medians and interquartile range (IQR) of the most abundant bacterial phyla (bold) and genera (italics) detected in the AS of either non-infected or infected bees collected in spring.

	Non-Infected		Infected		p-value
**Phylum/Genera**	**n (%)**	**Median (IQR)**	**n (%)**	**Median (IQR)**	
**Proteobacteria**	35 (100%)	83.7 (75.46-90.88)	36 (100%)	83.22 (74.61-93.59)	0.82
Snodgrassella	35 (100%)	25.7 (19.91-43.61)	36 (100%)	41.78 (21.65-51.76)	0.17
Gilliamella	35 (100%)	24.35 (15.56-35.97)	36 (100%)	21.4 (14.83-31.93)	0.39
Frischella	33 (94%)	4.29 (0.18-13.24)	29 (81%)	4.27 (0.27-11.95)	0.60
Pseudomonas	12 (34%)	<0.01 (<0.01-0.03)	11 (31%)	<0.01 (<0.01-0.03)	0.82
Bartonella	9 (26%)	<0.01 (<0.01-0.01)	6 (17%)	<0.01 (<0.01-<0.01)	0.34
Commensalibacter	7 (20%)	<0.01 (<0.01-<0.01)	6 (17%)	<0.01 (<0.01-<0.01)	0.60
Bombella	11 (31%)	<0.01 (<0.01-0.01)	16 (44%)	<0.01 (<0.01-0.06)	0.23
Enterobacter	5 (14%)	<0.01 (<0.01-<0.01)	2 (6%)	<0.01 (<0.01-<0.01)	0.25
Pantoea	6 (17%)	<0.01 (<0.01-<0.01)	6 (17%)	<0.01 (<0.01-<0.01)	0.91
Lonsdalea	0 (0%)	<0.01 (<0.01-<0.01)	0 (0%)	<0.01 (<0.01-<0.01)	not detected
Morganella	8 (23%)	<0.01 (<0.01-<0.01)	12 (33%)	<0.01 (<0.01-0.05)	0.42
**Firmicutes**	35 (100%)	13.4 (7.22-20.95)	36 (100%)	12.27 (5.56-22.8)	0.69
Lactobacillus	35 (100%)	12.24 (6.61-20.9)	36 (100%)	11.92 (5.48-19.95)	0.56
Fructobacillus	12 (34%)	<0.01 (<0.01-0.03)	12 (33%)	<0.01 (<0.01-0.07)	0.81
Leuconostoc	1 (3%)	<0.01 (<0.01-<0.01)	1 (3%)	<0.01 (<0.01-<0.01)	0.98
Spiroplasma	0 (0%)	<0.01 (<0.01-<0.01)	1 (3%)	<0.01 (<0.01-<0.01)	0.34
**Actinobacteriota**	33 (94%)	1.06 (0.55-2.43)	33 (92%)	0.67 (0.07-1.27)	**0.04**
Bifidobacterium	33 (94%)	1.06 (0.56-2.38)	31 (86%)	0.63 (0.04-1.25)	**0.041**
**Bacteroidota**	23 (66%)	0.02 (<0.01-0.05)	20 (56%)	0.01 (<0.01-0.07)	0.85
Dysgonomonas	4 (11%)	<0.01 (<0.01-<0.01)	1 (3%)	<0.01 (<0.01-<0.01)	0.18
Apibacter	4 (11%)	<0.01 (<0.01-<0.01)	6 (17%)	<0.01 (<0.01-<0.01)	0.64
**Acidobacteriota**	1 (3%)	<0.01 (<0.01-<0.01)	3 (8%)	<0.01 (<0.01-<0.01)	0.33
**Minor_phyla**	13 (37%)	<0.01 (<0.01-0.01)	11 (31%)	<0.01 (<0.01-<0.01)	0.63
Minor_genera	35 (100%)	0.14 (0.05-0.24)	34 (94%)	0.13 (0.05-0.3)	0.84
Unclassified_genera	35 (100%)	2.36 (0.96-7.33)	36 (100%)	2.28 (0.18-7.38)	0.73

*: Number of samples in which the phylum/genus was detected (relative frequency of detection).

† Wilcoxon rank sum test with Bonferroni correction

Supplementary Table 2. Relative frequencies, medians and interquartile range (IQR) of the most abundant bacterial phyla (bold) and genera (italics) detected in the AS of either non-infected or infected bees collected in autumn.

	**Non-Infected**		**Infected**		**p-value+**
Phylum/Genera	n (%)*	Median (IQR)	n (%)	Median (IQR)	
**Proteobacteria**	43 (100%)	75.61 (66.02-86.92)	44 (100%)	76.16 (64.73-81.39)	0.40
Snodgrassella	43 (100%)	22.22 (5.73-39.22)	44 (100%)	26.59 (13.84-41.45)	0.28
Gilliamella	43 (100%)	19.95 (12.41-32.24)	44 (100%)	15.89 (9.58-25.85)	0.31
Frischella	42 (98%)	9.04 (0.49-14.07)	44 (100%)	6.64 (0.95-13.39)	0.66
Pseudomonas	27 (63%)	0.01 (<0.01-0.09)	22 (50%)	<0.01 (<0.01-0.07)	0.22
Bartonella	13 (30%)	<0.01 (<0.01-0.02)	13 (30%)	<0.01 (<0.01-0.04)	0.85
Commensalibacter	20 (47%)	<0.01 (<0.01-0.04)	20 (45%)	<0.01 (<0.01-0.07)	0.90
Bombella	39 (91%)	0.08 (0.02-0.2)	34 (77%)	0.09 (0.01-0.44)	0.97
Enterobacter	10 (23%)	<0.01 (<0.01-<0.01)	11 (25%)	<0.01 (<0.01-<0.01)	0.78
Pantoea	10 (23%)	<0.01 (<0.01-<0.01)	9 (20%)	<0.01 (<0.01-<0.01)	0.71
Lonsdalea	17 (39.53%)	<0.01 (<0.01-0.05)	20 (45%)	<0.01 (<0.01-0.06)	0.82
Morganella	6 (13.95%)	<0.01 (<0.01-<0.01)	1 (2%)	<0.01 (<0.01-<0.01)	0.054
**Firmicutes**	43 (100%)	16.11 (8.55-24.79)	44 (100%)	19.02 (12.28-25.28)	0.33
Lactobacillus	43 (100%)	15.1 (7.99-24.75)	44 (100%)	17.03 (12.28-24.58)	0.34
Fructobacillus	14 (33%)	<0.01 (<0.01-0.02)	7 (16%)	<0.01 (<0.01-<0.01)	0.12
Leuconostoc	13 (30%)	<0.01 (<0.01-0.01)	15 (34%)	<0.01 (<0.01-0.02)	0.91
Spiroplasma	0 (0%)	<0.01 (<0.01-<0.01)	1 (2%)	<0.01 (<0.01-<0.01)	0.33
**Actinobacteriota**	43 (100%)	3.76 (2.05-6.88)	43 (98%)	5.76 (1.82-7.74)	0.42
Bifidobacterium	42 (98%)	3.76 (1.88-6.87)	43 (98%)	5.76 (1.81-7.74)	0.35
**Bacteroidota**	38 (88%)	0.06 (0.01-0.35)	36 (82%)	0.05 (0.01-0.94)	0.88
Dysgonomonas	11 (26%)	<0.01 (<0.01-0.01)	10 (23%)	<0.01 (<0.01-<0.01)	0.67
Apibacter	12 (28%)	<0.01 (<0.01-0.02)	15 (34%)	<0.01 (<0.01-0.05)	0.61
**Acidobacteriota**	12 (28%)	<0.01 (<0.01-0.01)	10 (23%)	<0.01 (<0.01-<0.01)	0.54
**Minor_phyla**	16 (37%)	<0.01 (<0.01-<0.01)	15 (34%)	<0.01 (<0.01-<0.01)	0.95
Minor_genera	37 (86%)	0.09 (0.01-0.92)	37 (84%)	0.15 (0.01-0.41)	0.67
Unclassified_genera	43 (100%)	1.85 (0.48-5.9)	44 (100%)	2.91 (0.86-10.47)	0.46

*: Number of samples in which the phylum/genus was detected (relative frequency of detection).

† Wilcoxon rank sum test with Bonferroni correction

Supplementary Table 3. Relative frequencies, medians and interquartile range (IQR) of the most abundant bacterial phyla (bold) and genera (italics) detected in the AS from younger (n= 58) and older (n = 100) bees.

	Younger		Older		p-value†
**Phylum/Genera**	**n (%)***	**Median (IQR)**	**n (%)***	**Median (IQR)**	
**Proteobacteria**	58 (100%)	78.8 (71.97-87.27)	100 (100%)	80.45 (67.29-89.87)	0.46
Snodgrassella	58 (100%)	38.17 (21.71-51.12)	100 (100%)	22.88 (11.15-41.02)	0.002
Gilliamella	58 (100%)	14.75 (7.09-21.73)	100 (100%)	25.26 (16.57-36.65)	<0.001
Frischella	58 (100%)	4.89 (0.91-13.57)	90 (90%)	6.17 (0.09-13.19)	0.40
Pseudomonas	29 (50%)	<0.01 (<0.01-0.05)	43 (43%)	<0.01 (<0.01-0.06)	0.81
Bombella	47 (81.03%)	0.1 (0.02-0.32)	53 (53%)	0.01 (<0.01-0.06)	<0.001
Bartonella	10 (17.24%)	<0.01 (<0.01-<0.01)	31 (31%)	<0.01 (<0.01-0.06)	0.044
Enterobacter	11 (18.97%)	<0.01 (<0.01-<0.01)	17 (17%)	<0.01 (<0.01-<0.01)	0.98
Pantoea	7 (12.07%)	<0.01 (<0.01-<0.01)	24 (24%)	<0.01 (<0.01-<0.01)	0.055
Lonsdalea	24 (41.38%)	<0.01 (<0.01-0.19)	13 (13%)	<0.01 (<0.01-<0.01)	<0.001
Morganella	3 (5.17%)	<0.01 (<0.01-<0.01)	24 (24%)	<0.01 (<0.01-<0.01)	0.003
Commensalibacter	11 (18.97%)	<0.01 (<0.01-<0.01)	42 (42%)	<0.01 (<0.01-0.03)	0.004
**Firmicutes**	58 (100%)	18.04 (7.98-25.73)	100 (100%)	13.99 (7.99-21.88)	0.073
Lactobacillus	58 (100%)	17.16 (7.9-23.88)	100 (100%)	13.07 (7.68-21.81)	0.17
Fructobacillus	43 (74.14%)	0.03 (<0.01-0.26)	2 (2%)	<0.01 (<0.01-<0.01)	<0.001
Leuconostoc	20 (34.48%)	<0.01 (<0.01-0.02)	10 (10%)	<0.01 (<0.01-<0.01)	<0.001
Spiroplasma	2 (3.45%)	<0.01 (<0.01-<0.01)	0 (0%)	<0.01 (<0.01-<0.01)	0.064
**Actinobacteriota**	56 (96.55%)	1.56 (0.09-3.23)	96 (96%)	2.25 (0.89-6.21)	0.033
Bifidobacterium	53 (91.38%)	1.27 (0.06-3.23)	96 (96%)	2.19 (0.83-6.21)	0.017
**Bacteroidota**	45 (77.59%)	0.02 (<0.01-0.06)	72 (72%)	0.06 (<0.01-0.92)	0.026
Apibacter	4 (6.9%)	<0.01 (<0.01-<0.01)	33 (33%)	<0.01 (<0.01-0.09)	<0.001
Dysgonomonas	3 (5.17%)	<0.01 (<0.01-<0.01)	23 (23%)	<0.01 (<0.01-<0.01)	0.003
**Acidobacteriota**	12 (20.69%)	<0.01 (<0.01-<0.01)	14 (14%)	<0.01 (<0.01-<0.01)	0.39
**Minor_phyla**	18 (31.03%)	<0.01 (<0.01-<0.01)	37 (37%)	<0.01 (<0.01-0.01)	0.24
Minor_genera	57 (98.28%)	0.2 (0.08-0.47)	86 (86%)	0.06 (0.01-0.3)	0.001
Unclassified_genera	58 (100%)	1.46 (0.18-7.58)	100 (100%)	2.56 (0.77-9.02)	0.16

*: Number of samples in which the phylum/genus was detected (relative frequency of detection).

† Wilcoxon rank sum test with Bonferroni correction
